# Toxic shock syndrome in the United States: surveillance update, 1979 1996.

**DOI:** 10.3201/eid0506.990611

**Published:** 1999

**Authors:** R A Hajjeh, A Reingold, A Weil, K Shutt, A Schuchat, B A Perkins

**Affiliations:** Centers for Disease Control and Prevention, Atlanta, Georgia 30333, USA. rfh5@cdc.gov

## Abstract

Menstrual toxic shock syndrome (TSS) emerged as a public health threat to women of reproductive age in 1979 80. We reviewed surveillance data for the period 1979 to 1996, when 5,296 cases were reported, and discuss changes in the epidemiologic features of TSS.


### Toxic Shock Syndrome in the United States: Surveillance Update, 1979-1996[1]

**Rana A. Hajjeh,* Arthur Reingold,† Alexis Weil,* Kathleen Shutt,* Anne Schuchat,* and Bradley A. Perkins***
*Centers for Disease Control and Prevention, Atlanta, Georgia, USA; and
†School of Public Health, University of California, Berkeley, California, USA

Menstrual toxic shock syndrome (TSS) emerged as a public health threat to women of reproductive age in 1979-80. We reviewed surveillance data for the period 1979 to 1996, when 5,296 cases were reported, and discuss changes in the epidemiologic features of TSS.

Toxic shock syndrome (TSS) emerged as a result of changes in industry and personal behavior but responded to rapid public health action, including active surveillance (1). This illness received national attention in 1980 when unexplained febrile illness associated with shock, multiorgan dysfunction, and high death rates was reported in healthy young women from several states (2,3). This clinical syndrome had been described sporadically since the 1920s (4). The dramatic increase in the number of cases in 1979-80 spurred epidemiologic, clinical, and laboratory studies that resulted in better understanding of the association between high-absorbency tampons and TSS (5). These studies led to recommendations that substantially decreased the risk for TSS (6,7).

TSS became a nationally notifiable disease in 1980 (8). After the initial epidemic, the number of reported cases decreased significantly. In 1986, active surveillance was conducted in many areas in the United States (total population 34 million) to confirm that trend (9). The cumulative incidence (0.5 per 100,000 population) confirmed the substantial decrease in the incidence of menstrual TSS observed in the passive surveillance system. Incidence rates decreased from 6 to 12 per 100,000 among women 12 to 49 years of age (10,11) in 1980 to 1 per 100,000 among women 15 to 44 years of age in 1986. These data also demonstrated that passive surveillance accurately described the demographic characteristics and case-fatality ratio of TSS cases (12,13).

Address for correspondence: Rana A. Hajjeh, Centers for Disease Control and Prevention, Division of Bacterial and Mycotic Diseases, 1600 Clifton Road, Mail Stop C09, Atlanta, GA 30333, USA; fax: 404-639-0817; e-mail: rfh5@cdc.gov.

Ongoing surveillance in the United States allows us to estimate the current incidence of TSS and monitor whether new menstrual or vaginal products affect the risk for disease. Other products, including high-absorbency disposable diapers, have raised similar questions regarding TSS in children. To address these questions and describe the current epidemiologic characteristics and recent temporal trends of the syndrome, we reviewed data from the ongoing national surveillance for TSS from 1979 through 1996, focusing on three periods: 1979 to 1980 (the epidemic years), 1981 to 1986 (a period of increased TSS awareness, culminating in active surveillance in 1986), and 1987 to 1996.

### The Study

The national TSS surveillance system has been described (8). Cases of TSS are reported to the Centers for Disease Control and Prevention (CDC) by state health departments in standardized case reports that include information on demographic and clinical characteristics, hospitalization status, outcome, laboratory data, products used during menses, and recurrence of menstruation-associated cases.

We used the surveillance case definition for TSS revised in 1981, which requires five clinical criteria: fever, hypotension, rash, desquamation, and abnormalities in three or more organ systems (8). A definite case fulfilled all five

[1]Presented in part at the European Conference on Toxic Shock Syndrome, Royal Society of Medicine, London, September 1997, and at the International Conference on Emerging Infectious Diseases, Atlanta, March 1998. (Arbuthnott J, Furman B, editors. European conference on toxic shock syndrome, 1997. London [UK]: The Royal Society of Medicine Press Limited; 1998.)

criteria (unless the patient died before desquamation), and a probable case fulfilled four of the five criteria. For the purposes of this analysis and because information on desquamation was often unavailable because of death or early discharge, we included all TSS reports meeting either definition. A similar change regarding exclusion of desquamation was adopted in a retrospective study (14).

A TSS case was considered menstrual if onset of symptoms occurred within 3 days of the beginning or end of menses; all other cases were considered nonmenstrual and were classified into three categories: surgical wounds, postpartum or postabortion, and other. Information on recurrent episodes of TSS was collected only from women with menstrual TSS, by asking them about a similar previous illness during menstruation. Data were analyzed by using SAS 6.1 (SAS, Cary, NC). The chi-square test was used to test for statistical significance.

From 1979 to 1996, 5,296 TSS cases were reported; 1,035 of these were reported from 1987 to 1996 (Figure). Overall, 93% of all TSS cases reported from 1979 to 1996 were among women. Although the proportion of menstrual cases decreased (Table), TSS affected mostly women. The median age was 22 years (3 days to 87 years); 91% were white. No significant seasonal variation was noted. For cases in which the source of the report was known (n = 2,118), 64% were reported by infection control practitioners and 28% by physicians.

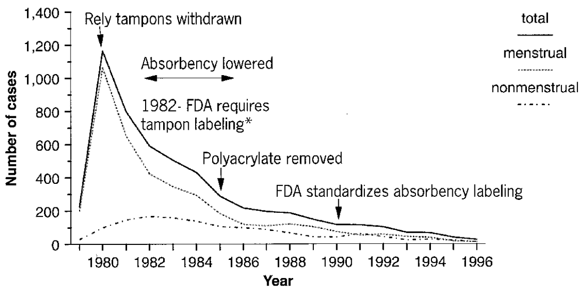

*FDA, Food and Drug Administration; includes definite and probable toxic shock syndrome cases

Figure. Toxic shock syndrome cases,* menstrual vs. nonmenstrual, United States, 1979–1996.

Menstrual TSS accounted for 74% of TSS cases during 1979 to 1996 (n = 5,296); this proportion, however, declined from 91% during 1979 and 1980 to 71% during 1981 to 1986 and 59% during 1987 to 1996. The median age of patients with menstrual TSS was 21 years, but 41% (n = 1,591) of menstrual TSS cases occurred in female patients 13 to 19 years of age. No significant change in the age distribution of menstrual cases was noted between the three periods (median age: 21 years, 1979 to 1980; 20 years, 1981 to 1986; 25 years, 1987 to 1996). Most (98%) patients with menstrual TSS cases for which the menstrual product was known (n = 3,457) reported tampon use, a proportion that did not change; 89% used tampons only, while 5.0% used both tampons and pads and 3% used tampons and minipads. The level of tampon absorbency was reported in only 41% (n = 1,385) of cases: 28% of patients used regular tampons, while 71% used super-absorbency tampons. *Staphylococcus aureus* was isolated from 90% of women with menstrual TSS who had vaginal cultures performed (n = 2,536). Of all the women with menstrual TSS, 1,606 (30%) responded to the question regarding recurrent disease, and 10% reported a previous illness similar to their TSS episode.

Nonmenstrual cases also occurred mostly in women (73%) and whites (87%). During 1987 to 1996, the proportion of all TSS cases that were

Table. Demographic characteristics, outcome, and proportion of menstrual cases of toxic shock syndrome, United States, 1979–1996

| | No. (%)[a] by years | | | |
|---|---|---|---|---|
| Characteristic | 1979-80 (n = 1,392) | 1981-86 (n = 2,835) | 1987-96 (n = 1,069) | Total cases (n = 5,296) |
| Female | 1,365 (98) | 2,594 (92) | 958 (90) | 4,917 (93) |
| Median age (yrs), (range) | 21 (1-70) | 22 (1-87) | 25 (3d-82) | 22 (3d-87) |
| White race | 1,067 (77) | 2,564 (90) | 967 (90) | 4,598 (91) |
| Menstrual cases | 1,264 (91) | 2,021 (71) | 636 (59) | 3,921 (74) |
| Deaths (Case-fatality ratio [%]) | 77 (6) | 95 (3.5) | 36 (3.5) | 208 (4) |

[a]Calculations were done by using as denominator the number of persons for whom the information on the specific characteristic was available.

nonmenstrual averaged 41% (30% to 55%). Of nonmenstrual cases, 18.3% were reported after surgical procedures, 11.5% were postpartum or postabortion, and 23.1% had nonsurgical cutaneous lesions. The proportion of all nonmenstrual cases reported after surgical procedures increased from 14% during 1979 to 1986 to 27% during 1987 to 1996 ($p < 0.05$). Among female nonmenstrual case-patients, 12% reported using barrier contraceptives (sponges and diaphragms); this proportion was, however, significantly less ($p < 0.05$) in 1987 to 1996 (6%) than in 1979 to 1986 (14%). Fifty cases of TSS in children $\leq$ 5 years of age were reported during the 17-year period; more than half of these occurred in children $\leq$ 2 years of age, and most (61.7%) were associated with nonsurgical cutaneous lesions. The percentage of TSS associated with nonsurgical cutaneous lesions (23.1%) was higher among younger patients (overall case-fatality ratio 4% [n = 2 deaths]) than among patients with other nonmenstrual cases.

Hospitalization status was known for 2,930 patients; 98% were hospitalized. The overall TSS case-fatality ratio was 4.1% (3% for menstrual cases, 5% for nonmenstrual cases) and was significantly higher among nonmenstrual cases ($p < 0.005$). Among menstrual cases, the case-fatality ratio decreased significantly with time (from 5.5% in 1979 and 1980, to 2.8% in 1981 to 1986, to 1.8% in 1987 to 1996, chi-square for linear trend [$p = 0.0001$]). This trend was not observed among nonmenstrual cases (for the three time periods: 8.5%, 5.3%, and 6%, respectively).

### Conclusions

Our review of recent passive surveillance data confirms the declining trend previously noted by active surveillance in 1986. Changes observed in the epidemiologic characteristics of TSS include an increase in the proportion of nonmenstrual cases and the difference in the risk for death between menstrual and nonmenstrual cases.

A number of factors could account for the observed decline, including the decrease in tampon absorbency, the standardized labeling required by the U.S. Food and Drug Administration; greater awareness of TSS among women; and the proliferation of educational materials for women, including tampon package inserts (12). However, at least 40% of menstrual TSS cases continue to affect women 13 to 19 years old, an age group not as likely to be aware of the risk for TSS and for whom further education may be needed.

Over the last few years, two changes have occurred in tampon use and composition: 1) All-cotton tampons have been introduced and marketed as an alternative product; in vitro studies have not, however, found any differences in the effects of these new tampons on the production of toxic shock syndrome toxin-1 (TSST-1) or its adsorption (15). 2) Tampons marketed specifically for overnight use have also been introduced. TSST-1 toxin production is not the only indicator of the potential risk for TSS. Previous case-control studies found that exclusive use of tampons was associated with a higher risk for TSS than use in conjunction with pads (13). Continued surveillance will monitor the effect of these changes on TSS occurrence. However, because the syndrome is rare, only large changes in the use of higher absorbency products would be likely to come to public health attention.

One of the important changes in the epidemiology of TSS is the increasing proportion of nonmenstrual cases, in particular, cases reported after surgical procedures. The factors contributing to this increase may include changes in delivery of surgical health-care services, with an increase in both outpatient procedures and the use of prosthetic devices. Hospitalizations caused by infections from prosthetic devices and postoperative infections increased significantly from 1980 to 1994 in the United States (16). In addition, the case-fatality ratio of nonmenstrual TSS cases did not decline, although the case-fatality ratio of menstrual TSS did. This difference in death rates could be due to several factors: nonmenstrual TSS may occur in less healthy (e.g., older) persons; diagnostic and reporting biases may result in more sensitive detection of severe cases of nonmenstrual TSS; decreased awareness of nonmenstrual TSS among health-care professionals may lead to increased deaths because of late treatment; and the nonspecific signs and symptoms of postoperative TSS may delay diagnosis (17). Further studies are needed to validate the difference in deaths between the two types of TSS and to clarify the risk factors for nonmenstrual TSS. Recent molecular studies of the gene that carries toxic shock toxin (18) may contribute to a better understanding of the emergence of TSS and transmission of toxin-producing strains of *S. aureus* and could improve prevention. Physicians, in particular surgeons, and other health-care professionals may need further education about the risks for nonmenstrual TSS.

Dr. Hajjeh is a medical epidemiologist in the Division of Bacterial and Mycotic Diseases Branch, CDC. Her areas of expertise and research interests include epidemiology of mycotic diseases, bacterial meningitis, and unexplained critical illnesses of possible infectious etiology.

### References

1. U.S. Public Health Services. Addressing emerging infectious disease threats. A prevention strategy for the United States. Atlanta: Centers for Disease Control and Prevention; 1994.
2. Todd JK, Kapral FA, Fishaut M, Welch TR. Toxic shock syndrome associated with phage group 1 staphylococci. Lancet 1978;2:1116-8.
3. Centers for Disease Control and Prevention. Toxic-shock syndrome—United States. MMWR Morb Mortal Wkly Rep 1980;29:229-30.
4. Broome CV. Epidemiology of toxic shock syndrome in the United States: Overview. Reviews of Infect Diseases 1989:2 Suppl 1:S14-21.
5. Shands KN, Schmid GP, Dan BB, Blum D, Guidotti RJ, Hargrett NT, et al. Toxic-shock syndrome in menstruating women: association with tampon use and *Staphylococcus aureus* and clinical features in 52 cases. N Engl J Med 1980:303:1436-42.
6. Centers for Disease Control and Prevention. Toxic shock syndrome—United States. MMWR Morb Mortal Wkly Rep 1980:297–9.
7. Centers for Disease Control and Prevention. Follow-up on toxic-shock syndrome. MMWR Morb Mortal Wkly Rep 1980;29:441-5.
8. Reingold AL, Hargrett NT, Shands KN, Dan BB, Schmid GP, Strickland BY, et al. Toxic shock syndrome surveillance in the United States, 1980 to 1981. Ann Intern Med 1982;96 (part 2):875-80.
9. Gaventa S, Reingold AL, Hightower AW, Broome CV, Schwartz B, Hoppe C, et al. Active surveillance for toxic shock syndrome in the United States. 1986. Reviews of Infectious Diseases 1989:2 Suppl 1:S28-34.
10. Davis JP, Chesney PJ, Wand PJ, LaVenture M. Toxic-shock syndrome: epidemiologic features, recurrence, risk factors and prevention. N Engl J Med 1980:303:1429-35.
11. Latham RH, Kehrberg MW, Jacobson JA. Toxic shock syndrome in Utah: a case-control and surveillance study. Ann Intern Med 1982:96:906-8.
12. Schuchat A, Broome CV. Toxic shock syndrome and tampons. Epidemiol Rev 1991:13:99-112.
13. Centers for Disease Control and Prevention. Reduced incidence of menstrual toxic shock syndrome—United States, 1980-1990. MMWR Morb Mortal Wkly Rep 1990;39:421-4.
14. Petitti DB, Reingold AL. Update through 1985 on the incidence of toxic shock syndrome among members of a prepaid health plan. Reviews of Infectious Diseases 1989;11 Suppl 10:S22-7.
15. Schlievert PM. Comparison of cotton and cotton/rayon tampons for effect on production of toxic shock syndrome toxin. J Infect Dis 1995;172:1112-4.
16. Simonsen L, Conn LA, Pinner RW, Teutsch S. Trends in infectious disease hospitalizations in the United States, 1980-1994. Arch Intern Med 1998;158:1923-8.
17. Hughes D, Stapleton J. Postoperative toxic shock syndrome. Iowa Med 1991;81:55-8.
18. Lindsay J, Ruzin A, Ross HF, Kurepina N, Novick RP. The gene for toxic shock toxin is carried by a family of mobile pathogenicity islands in *Staphylococcus aureus*. Mol Microbiol 1998;29:527-43.

